# Chemical and electrochemical lithiation of van der Waals oxytelluride V_2_Te_2_O†

**DOI:** 10.1039/d5dt00159e

**Published:** 2025-02-21

**Authors:** Nicola D. Kelly, Heather Grievson, Katherine M. Steele, Ivan da Silva, Simon J. Clarke

**Affiliations:** a Department of Chemistry, University of Oxford, Inorganic Chemistry Laboratory South Parks Road Oxford OX1 3QR UK simon.clarke@chem.ox.ac.uk; b The Faraday Institution Quad One Harwell Campus Didcot OX11 0RA UK; c School of Chemical, Materials and Biological Engineering, University of Sheffield Sir Robert Hadfield Building Sheffield S1 3JD UK; d ISIS Neutron and Muon Source, STFC, Rutherford Appleton Laboratory Harwell Campus Didcot OX11 0QX UK

## Abstract

Reversible lithium intercalation into the van der Waals phase V_2_Te_2_O, forming new phases Li_*x*_V_2_Te_2_O with *x* approaching 2, is reported using both chemical and electrochemical methods. The progress of each reaction was followed using powder X-ray diffraction and the crystal structure of the intercalated phase with *x* = 1, LiV_2_Te_2_O, was refined using powder neutron diffraction. The intercalated Li ions occupy vacant pseudo-octahedral sites and the unit cell expands on reduction with no change in symmetry. The lithium ions can be removed chemically or electrochemically, making this the first known oxytelluride to undergo reversible lithium intercalation.

## Abstract 2

Reversible intercalation of lithium into the layered oxytelluride V_2_Te_2_O using both chemical and electrochemical methods is demonstrated.

## Graphical abstract

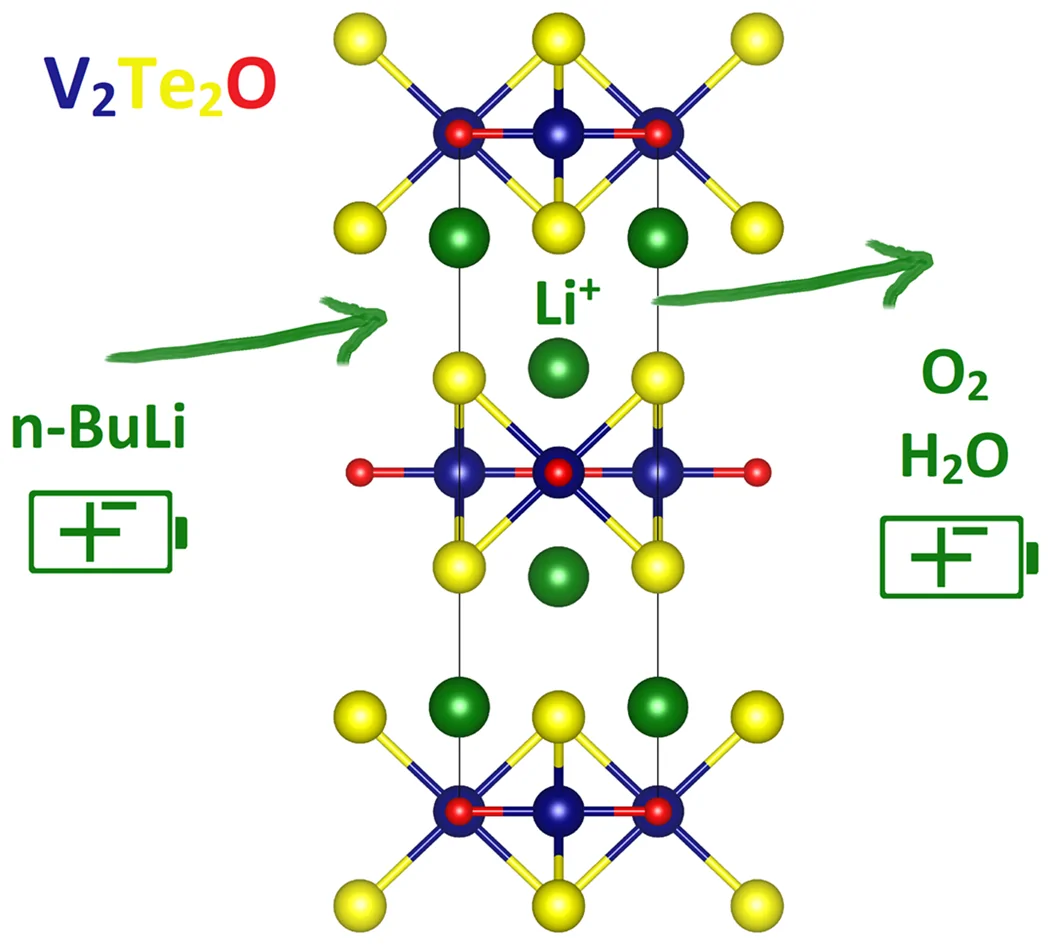

## Introduction

Intercalation of alkali metal ions into layered transition-metal compounds is integral to the operation of rechargeable batteries. Much current materials chemistry research is focused on optimising these cathode materials for efficiency, cost, scale and reversibility considerations.^1^ A persistent problem is the high cost of cobalt, a key ingredient in many commercial Li-ion batteries with LiCoO_2_ as the cathode. Ni and Mn substitution have been explored to mitigate this problem^2^ but systems based on entirely different transition metal ions are also being developed, with a particular focus on iron-based oxides,^3^ sulphates^4^ and phosphates,^5^ since iron is a light and naturally very earth-abundant transition metal. These properties make iron-based cathodes attractive because of their high energy density and low cost respectively.

Vanadium is lighter than iron and more abundant in the continental crust than cobalt, nickel or copper.^6^ Furthermore, it has many stable positive oxidation states (+2 to +5 inclusive), enabling multi-electron redox activity and hence a high theoretical capacity for rechargeable batteries.^7,8^ Recent studies on vanadium-based cathodes show promising results for polyanionic compounds, with reversible cycling observed for Li_2_VO(SO_4_)_2_,^9^ Na_4_VO(PO_4_)_2_,^10^ NaVPO_4_F,^11^ ε-VOPO_4_,^12^ and α- and β-VOSO_4_ ^13^ among others.

Vanadium oxytelluride, V_2_Te_2_O, was first synthesised in 2018 by Ablimit *et al.* by reacting RbV_2_Te_2_O with H_2_O at room temperature to extract the Rb^+^ ions.^14^ It can similarly be produced by deintercalation of K^+^ ions from KV_2_Te_2_O.^15^ V_2_Te_2_O consists of square planar layers of V^3+^ and O^2−^ ions, sandwiched between Te^2−^ layers which are weakly bonded in the *c* direction by van der Waals interactions. Similar van der Waals gaps are known to accept intercalated metal ions in layered tellurides including VTe_2_,^16^ NbTe_2_ and TaTe_2_,^17^ Ti_2_PTe_2_ and ZrPTe_2_,^18^ Bi_2_Te_3_,^19^ CrGeTe_3_,^20^ Fe_3_GeTe_2_ ^21^ and Fe_5_GeTe_2_,^22^ sometimes leading to dramatic physical property changes, such as an increase in the Curie temperature of CrGeTe_3_ from 66 to 240 K with Na insertion. However, to our knowledge no such investigations have been carried out on oxytellurides, *i.e.*, compounds containing both Te^2−^ and O^2−^ anions. Therefore, despite the presence of scarce, heavy and toxic Te atoms making it unviable for commercial battery applications, V_2_Te_2_O presents a worthwhile platform for investigating the fundamental chemistry and physics of lithium intercalation and property tuning *via* controlling the electron count. In this article we report the reductive intercalation of Li^+^ ions into V_2_Te_2_O both chemically, using *n*-butyllithium solution, and electrochemically, in cells where a Li metal anode is the Li source. The body-centred tetragonal symmetry of the host material is preserved upon insertion of Li^+^. The chemically lithiated products undergo oxidative deintercalation by air or water to re-form V_2_Te_2_O; our electrochemical studies also indicate the reversibility of Li insertion.

## Experimental methods

### Synthesis

All reagents and samples were treated as air- and moisture-sensitive and were therefore handled and manipulated in an argon-filled glovebox. The host compound V_2_Te_2_O was prepared from KV_2_Te_2_O as reported previously.^15^ Briefly, KV_2_Te_2_O samples each of target mass 1.0 g were prepared from a stoichiometric mixture of K metal (99.95%, Alfa Aesar) with powdered V_2_O_5_ (99.995%, Alfa Aesar), V (99.95%, Alfa Aesar) and Te (99.999%, Alfa Aesar). The reagents were placed in alumina crucibles, vacuum-sealed into silica ampoules and heated to 300 °C for 12 h then 700 °C for 24 h with intermediate grinding. For each step, the ramp rates were 1 °C min^−1^ on heating and the natural rate of the furnace on cooling. Subsequently, using a Schlenk line apparatus, degassed, deionised H_2_O (50 cm^3^) was added to ∼1 g KV_2_Te_2_O powder under a nitrogen atmosphere and stirred overnight at room temperature. Filtration, washing with tetrahydrofuran (2 × 20 cm^3^) and drying under dynamic vacuum yielded the target compound as a black powder.

Chemical lithiation was carried out on a Schlenk line. Typically, 0.1–0.2 g of V_2_Te_2_O powder was placed in a Schlenk flask under nitrogen and suspended in 5–10 cm^3^ of dry hexane. (The sample for neutron diffraction was synthesised in one batch on a larger scale, using 0.65 g of V_2_Te_2_O and 20 cm^3^ hexane.) An appropriate stoichiometric amount of *n*-butyllithium (1.6 M in hexanes, Sigma-Aldrich) was injected into the flask and the black suspension was stirred overnight at room temperature under a nitrogen atmosphere. The sample was then filtered, washed twice with hexane, and dried under dynamic vacuum to leave a fine black powder. Subsequent delithiation reactions were also carried out on the Schlenk line using water as the oxidant. 5–10 cm^3^ of deionised H_2_O was introduced into a Schlenk flask containing Li_*x*_V_2_Te_2_O under nitrogen followed by stirring overnight, filtering, washing with tetrahydrofuran and drying under vacuum as described above.

### Diffraction measurements

Laboratory powder X-ray diffraction (PXRD) for phase identification was carried out at room temperature on either a PANalytical Empyrean or a Bruker Avance Eco D8 diffractometer, both using CuKα radiation, *λ* = 1.541 Å, and with the former instrument equipped with a monochromator to select only CuKα_1_ radiation. Powder samples were loaded onto a lightly greased glass slide, mounted in a custom-made airtight sample holder with Mylar® windows and measured in the range 5 ≤ 2*θ* (°) ≤ 70. Cycled cathode films were attached to the airtight holder using Kapton® tape around the edges, avoiding covering the area of material being illuminated by the X-ray beam.

Synchrotron PXRD for accurate and precise structural refinement was carried out at room temperature at the I11 beamline,^23^ Diamond Light Source, Didcot, UK, with a wavelength of *λ* ≈ 0.82 Å (calibrated precisely at the start of each beamtime session using a Si standard). Samples were mixed 1 : 1 by volume with ground glass to reduce beam absorption and preferred orientation and packed into borosilicate capillaries of inner diameter 0.5 mm. Diffraction patterns were collected using a Mythen Position Sensitive Detector (PSD).

Time-of-flight powder neutron diffraction (PND) was carried out at room temperature on the GEM instrument at the ISIS Neutron and Muon Source, Oxfordshire, UK.^24^ The sample of mass 0.35 g was packed into a 6 mm vanadium canister and sealed with an indium gasket under inert atmosphere.^25^ Structure solution and Rietveld refinement^26^ were carried out using TOPAS-Academic V7.^27^ VESTA^28^ was used for crystal structure visualisation and production of figures.

### Electrochemical measurements

The active material (V_2_Te_2_O) was mixed with polyvinylidene fluoride (MTI Corporation) and carbon black C-NERGY Super C65 (Imerys Graphite & Carbon) in a mass ratio of 8 : 1 : 1 and ground by hand for 15 minutes then made into a slurry with *N*-methyl pyrrolidone (Merck). The slurry was cast onto carbon-coated aluminium foil and dried at 80 °C in air then in an 80 °C vacuum oven for at least 16 h. CR2032 SS316 coin cells (Cambridge Energy Solutions Ltd) were assembled in an inert atmosphere glove box using 12 mm diameter cathodes cut from the cast film, 16 mm diameter separators of Whatman glass microfibre (Merck), 15.6 mm diameter lithium metal chips (Cambridge Energy Solutions Ltd) as the anode, and for the electrolyte 1 M LiPF_6_ in ethylene carbonate and ethyl methyl carbonate, 3 : 7 v/v (Solvionic). Cyclic voltammetry was conducted at room temperature using a Biologic VMP-300 potentiostat and galvanostatic cycling was conducted at 25 °C using a MACCOR Series 4000. The cycling rate was *C*/20, where *C* is the theoretical capacity of the compound and *C*/20 means fully charging or discharging over 20 hours.

## Results and discussion

### Synthesis of V_2_Te_2_O

The host compound, produced from KV_2_Te_2_O using the soft-chemical method described above, was an air-stable black powder. The chemical yield was calculated using the mass of product; it varied between 75–85%, reflecting some loss of powder upon filtration and extraction of the product from the Schlenk flask. Rietveld refinement of PXRD data indicated that V_2_Te_2_O crystallises in the body-centred tetragonal space group *I*4/*mmm* as reported previously.^14^ The crystal structure, shown in Fig. 1(a), comprises planar V_2_O layers in the ab plane, composed of vertex-linked OV_4_ squares, sandwiched between two layers of telluride ions so that each vanadium ion forms a *trans*-{VO_2_Te_4_} distorted octahedron. The resulting neutral Te–V_2_O–Te slabs are stacked in a staggered manner along the *c* direction with van der Waals interactions between adjacent Te layers. A representative Rietveld refinement against synchrotron data is given in Fig. 1(b); the refined lattice parameters for this sample were *a* = 3.92667(2) Å, *c* = 13.2181(2) Å. Samples reproducibly had high phase purity, containing less than 5 wt% each of the secondary phases KV_3_Te_3_O_0.33_ ^29^ and/or V_2_O_3_. These secondary phases are often produced during the initial ceramic synthesis and they remain in the sample after reaction with water.

**Fig. 1:**
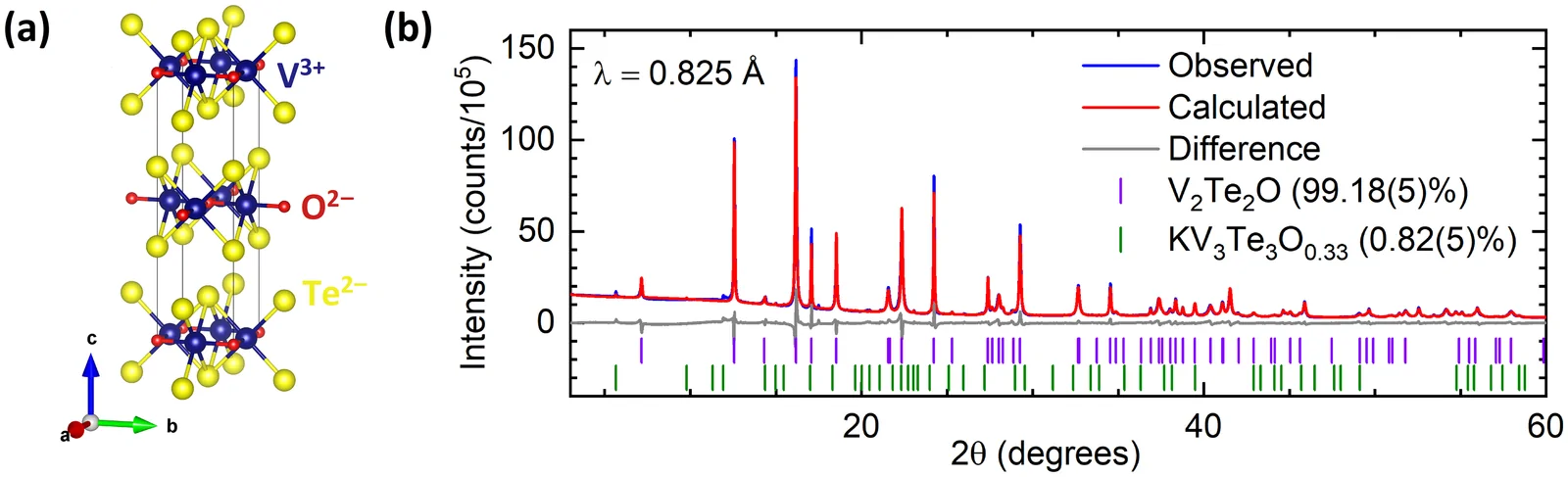
(a) Crystal structure of V_2_Te_2_O^14^ in tetragonal space group *I*4/*mmm*. (b) Rietveld refinement of synchrotron PXRD data for V_2_Te_2_O at room temperature. Blue – experimental data; red – calculated intensities; grey – difference pattern; tick marks – Bragg reflection positions for V_2_Te_2_O (purple) and KV_3_Te_3_O_0.33_ (green).

### Chemical lithiation and powder X-ray diffraction

V_2_Te_2_O was lithiated using solutions of *n*-butyllithium (*n*BuLi), resulting in an expansion of the unit cell in both *a* and *c* directions as observed by shifting of peaks in the PXRD patterns towards lower angles. This behaviour is consistent with the insertion of Li^+^ ions and electrons causing reduction of the vanadium ions and hence lengthening of the V–O and V–Te bonds. Fig. 2 shows the resultant PXRD patterns for several different reaction stoichiometries. The patterns for the intercalated samples all have broader peaks than the parent material, indicating lower crystallinity, particularly in samples made with smaller amounts of *n*BuLi.

**Fig. 2:**
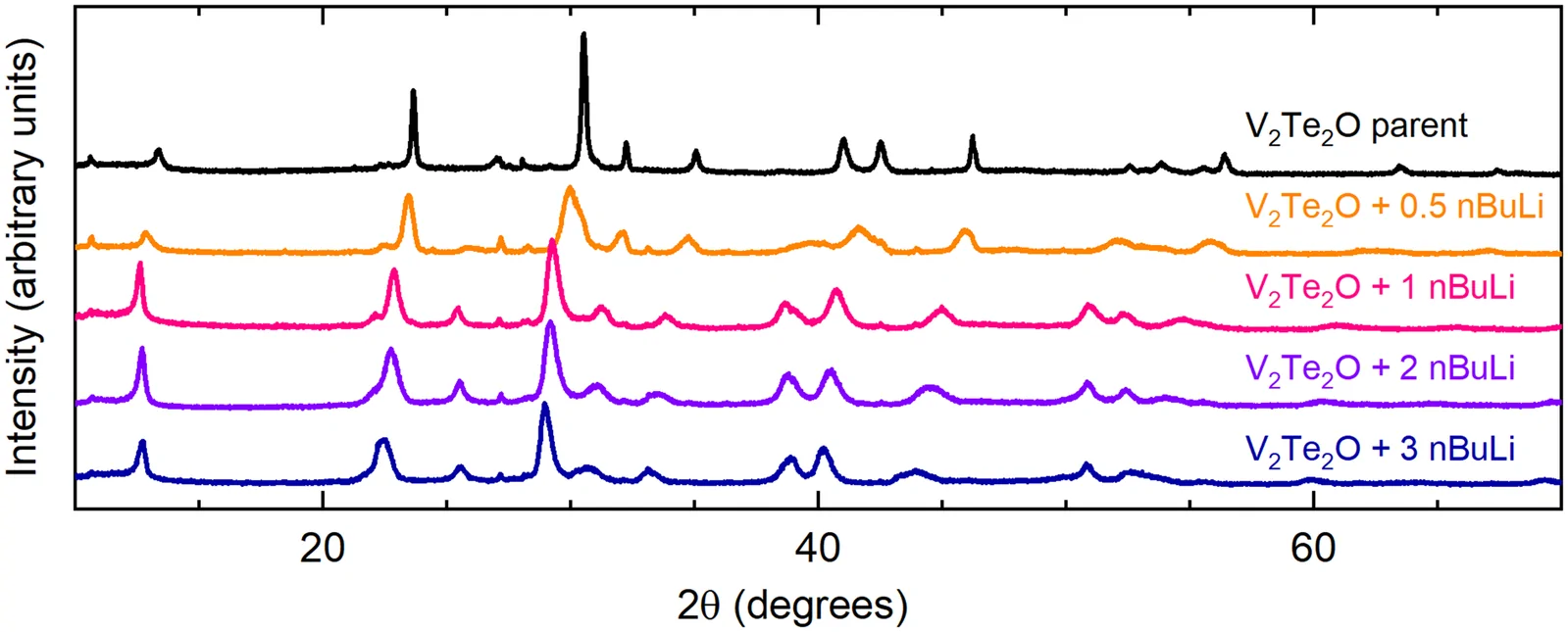
Laboratory PXRD patterns for the results of the reaction V_2_Te_2_O + *x n*BuLi, where 0 ≤ *x* ≤ 3 (top to bottom).

Rietveld refinement was carried out on PXRD data for all of the intercalated samples using the structure of V_2_Te_2_O^14^ with an expanded unit cell. The positions of the V and O atoms at (½, 0, 0) and (0, 0, 0) respectively and the *x* and *y* coordinates of the Te atoms (special positions) at (½, ½, *z*Te) were kept fixed while the *z*-coordinate of Te was refined. Owing to their low X-ray scattering power, Li atoms were not included in the structural model for refinements against X-ray data. Fig. 3 shows a representative Rietveld refinement of synchrotron PXRD data for an intercalated phase.

**Fig. 3:**
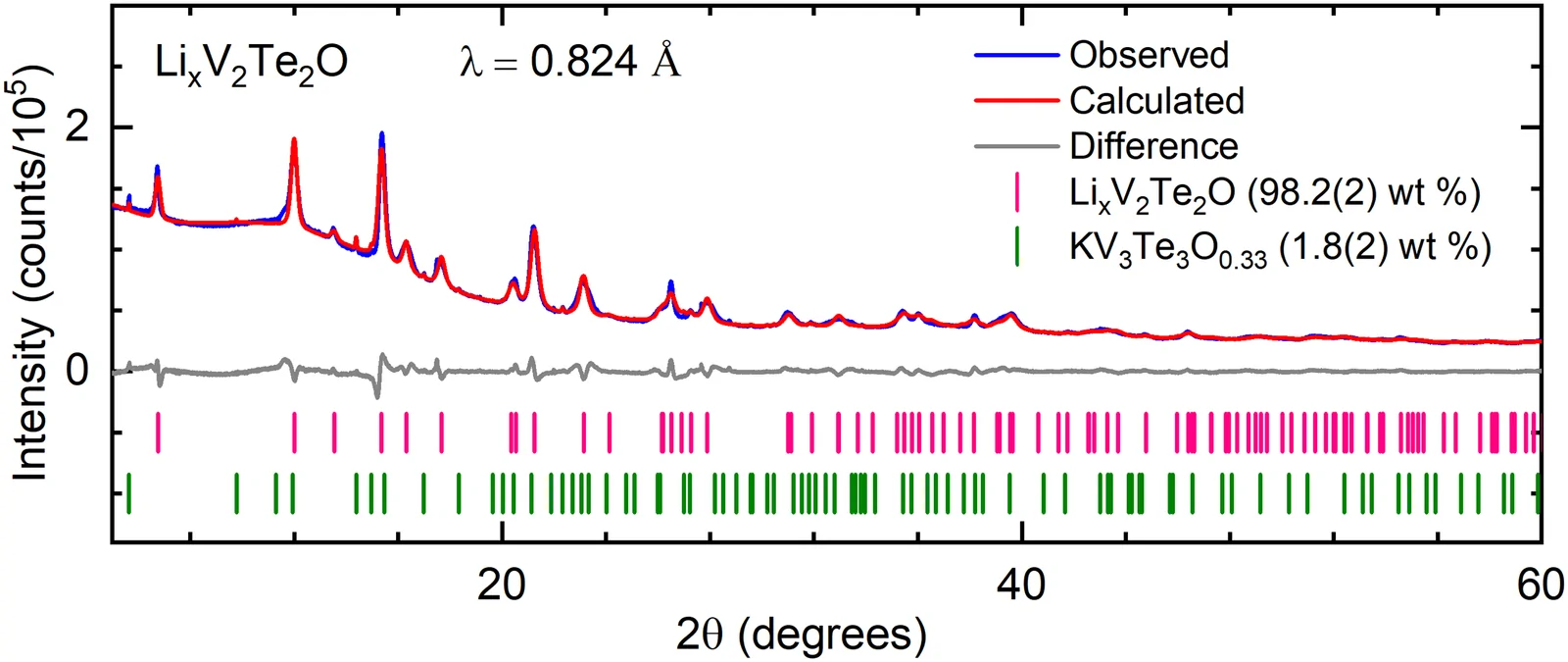
Rietveld refinement of synchrotron PXRD data for Li_*x*_V_2_Te_2_O (synthesised using 2 molar equiv. *n*BuLi) at room temperature. Blue – experimental data; red – calculated intensities; grey – difference pattern; tick marks – Bragg reflection positions for Li_*x*_V_2_Te_2_O (pink) and KV_3_Te_3_O_0.33_ (green).

The refined lattice parameters from laboratory PXRD data for a series of intercalated phases are given in Table 1 and Fig. 4, which plots the parameters relative to the parent material V_2_Te_2_O. The expansion in *c* increases upon reaction with up to 1 equivalent of *n*BuLi, then plateaus at approximately 5%, whereas the expansion in *a* increases steadily until 3–4 molar equivalents of *n*BuLi and then decreases again. We suggest that this behaviour is consistent with insertion of Li^+^ into the van der Waals gaps of V_2_Te_2_O, which consists of layers perpendicular to the *c* axis. The increase in *c*, corresponding to the interlayer spacing, would be approximately the same for any number of intercalated Li^+^ ions per formula unit above a certain threshold, because Li^+^ has a finite size. From the data, this threshold appears to be approximately 1–1.5 Li ions per formula unit of V_2_Te_2_O. By contrast, the *a* parameter could continue to increase as more Li is are inserted because it depends on the V–O bond length, which will increase as vanadium is reduced. From the information in Fig. 4, the limit of Li intercalation appears to be reached after reaction with 3–4 equivalents of *n*BuLi. After this point, the use of excess *n*BuLi favours the production of secondary impurity phases (evident in the powder diffraction patterns – see Fig. S3 in the ESI†). This leaves less Li available to form the main Li_*x*_V_2_Te_2_O phase, hence the subsequent decrease in the lattice parameters upon adding excess *n*BuLi (*i.e.* the behaviour of the lattice parameters shows that the intercalates obtained with excess *n*BuLi are actually less Li-rich than those obtained with a modest excess). We attempted to suppress side reactions by reducing the reaction time and/or temperature, but this resulted in no Li_*x*_V_2_Te_2_O intercalate being formed.

**Fig. 4:**
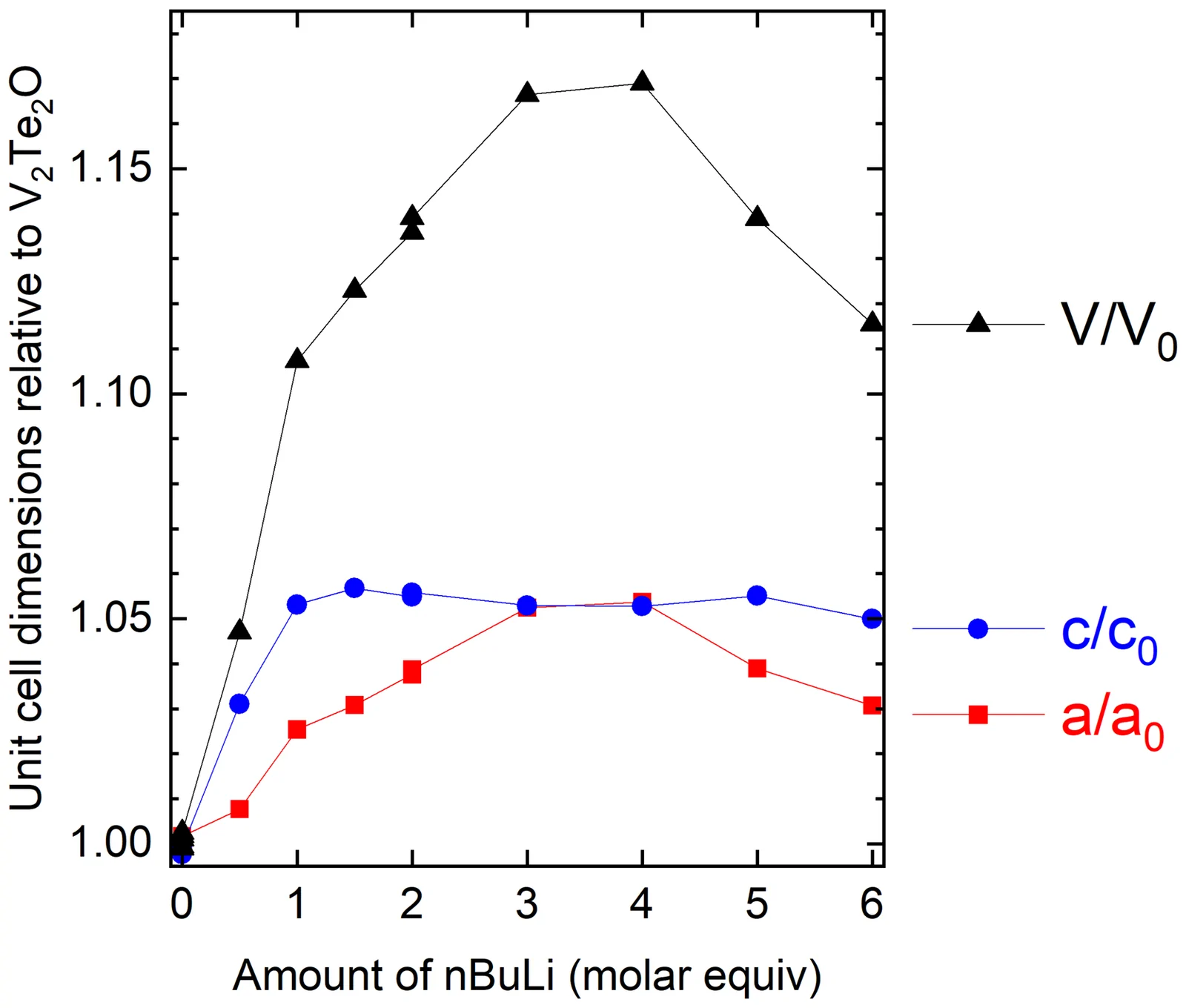
Refined lattice parameters for Li-intercalated samples, relative to the published parameters for V_2_Te_2_O,^14^ as a function of reaction stoichiometry. Side reactions at high Li stoichiometries in the reaction result in less Li-rich intercalates with smaller cell volumes than the maximum.

**Table 1:** Refined lattice parameters for chemically intercalated samples Li_*x*_V_2_Te_2_O from Rietveld refinement of laboratory PXRD data at room temperature. Space group: *I*4/*mmm* (no. 139). Note that the number of moles of *n*BuLi used in the reaction is not equal to the number of moles of Li intercalated into the host as discussed in the text

Sample code	Moles *n*BuLi in reaction mixture	*a* (Å)	*c* (Å)	Volume (Å^3^)
V_2_Te_2_O in literature^14^	—	3.9282(1)	13.2477(5)	204.42(2)
V_2_Te_2_O NDK235	—	3.9347(2)	13.2371(10)	204.93(3)
NDK304	0.5	3.9586(7)	13.659(3)	214.05(10)
NDK187	1.0	4.0280(7)	13.952(4)	226.36(10)
NDK305	1.5	4.0493(6)	14.000(3)	229.56(8)
NDK203	2.0	4.0761(5)	13.975(3)	232.19(8)
NDK260^a^	2.0	4.0803(10)	13.987(4)	232.9(2)
NDK204	3.0	4.1344(6)	13.949(2)	238.44(9)
NDK303	4.0	4.1392(9)	13.947(4)	239.0(2)
NDK341	5.0	4.0812(9)	13.978(4)	232.8(2)
NDK353	6.0	4.0488(8)	13.910(4)	228.0(2)

a Sample used for neutron powder diffraction.

Since the lowest non-zero stable oxidation state for vanadium is +2, V_2_Te_2_O is highly unlikely to accommodate insertion of more than two Li per formula unit, so there is likely an offset in the *x*-axis between the amount of BuLi added and the actual stoichiometry due to the side reactions. The limiting composition is discussed further below in the context of additional results from powder neutron diffraction and electrochemical cycling.

### Structure solution using powder neutron diffraction

The crystal structure of a 0.35 g sample of Li_*x*_V_2_Te_2_O, synthesised using 2 molar equivalents of *n*BuLi in the reaction, was investigated using time-of-flight PND at room temperature. We first examined the GEM bank 2 data because it has sufficient range in *d*-spacing to include the (002) reflection at ∼7 Å while also providing sufficient resolution for structural analysis. For the initial structural model we again used the published crystal structure of V_2_Te_2_O^14^ with an expanded unit cell; the initial lattice parameters were taken from PXRD analysis on the same sample and then refined against the neutron data using the Pawley method.^30^ In the subsequent Rietveld refinement there were discrepancies between the calculated and observed intensities for several Bragg reflections, with a noticeable over-estimate of the (110) peak intensity. Li ions (Li has a negative scattering length^31^) were added to the model at sites (0, 0, *z*Li) where the starting value for *z*Li was chosen as 0.24 (approximately in the middle of the van der Waals gaps). Refining the Li and Te *z*-coordinates and the Li fractional site occupancy occLi (limited to values between 0 and 1 inclusive, where full occupancy would correspond to *x* = 2 in the formula Li_*x*_V_2_Te_2_O) resulted in an adequate fit with *z*Li ∼0.15, *z*Te ∼0.14, and occLi ∼0.5. We also attempted to model lithium in tetrahedral sites in the van der Waals gaps, (½, 0, ¼), but this did not fit the observed data as the Li occupancy refined to zero.

We subsequently carried out a combined Rietveld refinement on the data from GEM banks 2, 3 and 4. Owing to the low data quality, reflecting the small sample size, the high absorption cross-section of Li^31^ and the incoherent background from V, a single isotropic displacement parameter *B*_iso_ was refined for all 4 elements. The refined crystal structure and the bank 3 data are shown in Fig. 5; the other banks are shown in the ESI (Fig. S1 and S2†). The refined structural parameters are given in Table 2.

**Fig. 5:**
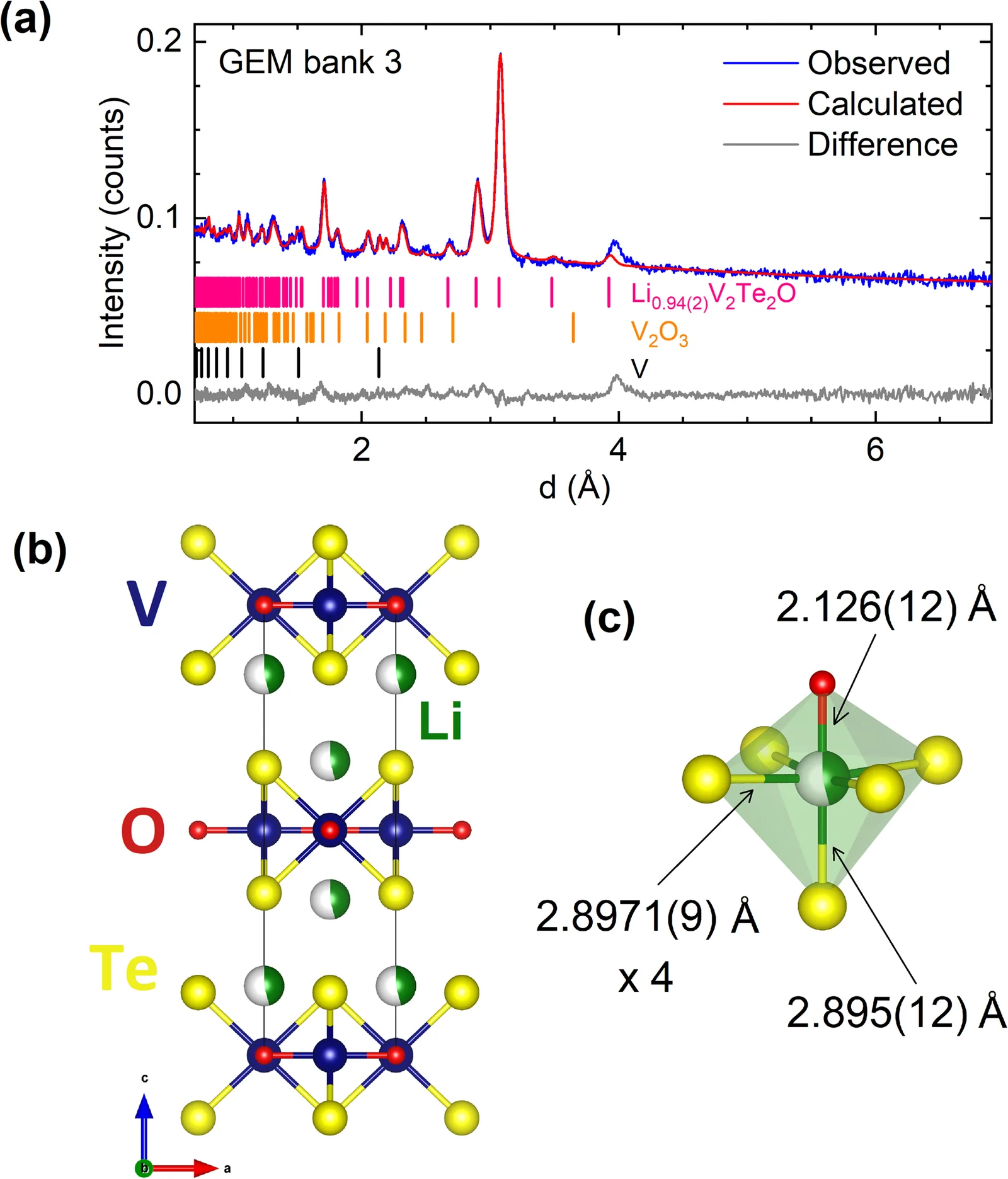
(a) Rietveld refinement of room temperature PND data for Li_*x*_V_2_Te_2_O measured on bank 3 of the GEM diffractometer at ISIS. Blue – experimental data; red – calculated intensities; grey – difference pattern; tick marks – Bragg reflection positions for Li_*x*_V_2_Te_2_O (pink), V_2_O_3_ (orange) and V sample container (black). (b) Structural model viewed perpendicular to the *c* axis. (c) Lithium-centred polyhedron.

Structural parameters for Li_*x*_V_2_Te_2_O (sample NDK260) at room temperature, determined from a combined refinement of neutron time-of-flight powder diffraction data from GEM banks 2, 3 and 4

**Table:** 

Composition	Li_0.94(2)_V_2_Te_2_O
Space group	*I*4/*mmm* (#139)
*a* (Å)	4.0887(6)
*c* (Å)	13.921(3)
Bank 2	*R*_wp_ = 3.22%, *χ*^2^ = 1.10
Bank 3	*R*_wp_ = 2.49%, *χ*^2^ = 2.31
Bank 4	*R*_wp_ = 2.11%, *χ*^2^ = 3.14

**Table:** 

Atom	Site	*x*	*y*	*z*	Fractional occupancy	*B*_iso_ (Å^2^)
Li	4e	0	0	0.1527(8)	0.471(9)	0.19(2)
V	4c	½	0	0	1	
Te	4e	½	½	0.1394(2)	1	
O	2a	0	0	0	1	

The refinement (Fig. 5(a) and ESI, Fig. S1 and S2†) includes two clear unindexed peaks at *d* ∼4.0 and 2.5 Å likely resulting from one or more impurity phases. The presence of impurities is perhaps unsurprising given the excess of *n*BuLi used compared with the final stoichiometry, with the remaining *n*BuLi presumably having been used up in side reactions. Furthermore, a peak at 4 Å is clearly visible in laboratory and synchrotron X-ray data for the majority of our *n*BuLi-intercalated samples (see *e.g.*Fig. 3 at 2*θ* ∼10°) and its intensity relative to the main phase increases with increasing amounts of *n*BuLi (ESI, Fig. S3†). However, the additional intensity could not be fitted by any appropriate binary or ternary compound in the Inorganic Crystal Structure Database (ICSD). We also attempted to fit a second Li_*x*_V_2_Te_2_O phase with either an expanded body-centred structure, or a primitive tetragonal structure similar to that of KV_2_Te_2_O,^15^ but neither model was able to account satisfactorily for the impurity peaks.

The intercalated lithium ions occupy pseudo-octahedral sites (Fig. 5(c)) where each Li ion is bonded to one oxide anion at a distance of 2.13(2) Å and five telluride anions at distances of 2.8971(9) Å (equatorial) and 2.90(2) Å (axial). These values are consistent with Li–O and Li–Te bond lengths in octahedral environments in compounds reported in the literature, *e.g.* 2.12 Å in LiVO_2_ ^32^ and 2.93 Å in LiCrTe_2_.^33^ This site is also chemically reasonable, considering lithium's strong affinity for oxygen according to HSAB theory.

The refined site occupancy for Li of 0.471(9) corresponds to the composition Li_0.94(2)_V_2_Te_2_O and an average vanadium oxidation state of +2.53, close to the oxidation states observed in the related 1221 phases A_1−*δ*_V_2_Te_2_O (A = K, Rb, Cs).^15,34^ The refined bond length for V–O (equivalent to *a*/2) in our sample is 2.0443(4) Å; for comparison, the V–O bond lengths for 6-coordinate vanadium(iii) are 1.968 Å in V_2_O_3_ ^35^ and 1.9641(1) Å in V_2_Te_2_O,^14^ whereas the vanadium(ii) oxide VO has bond lengths of 2.06 Å.^36^ Therefore, our result is consistent with an average vanadium oxidation state between +2 and +3 in Li_0.94_V_2_Te_2_O. Furthermore, the V–Te bond length from our neutron refinement is 2.8183(15) Å, which is very similar to the 2.798 Å of Rb_1−*δ*_V_2_Te_2_O with almost the same vanadium oxidation state.^34^

### Chemical delithiation

Lithium ions could be extracted from Li_*x*_V_2_Te_2_O upon exposure to moist air. A sample with *x* = 0.94, as determined by powder neutron diffraction, was investigated by first carrying out a 5-minute laboratory PXRD scan over a narrow 2*θ* range, 10–15°, to encompass only the (002) reflection. The airtight lid of the sample holder was then removed for 2 seconds to introduce air, the lid was replaced and a further nine such scans were collected in succession. Fig. 6 shows the background-subtracted data which demonstrate a shift to higher angles (smaller *d*-spacing) with increasing time, indicating the removal of Li^+^ ions and re-oxidation of vanadium. After 24 hours the sample had fully decomposed with V_2_Te_2_O and elemental Te evident in the powder pattern (ESI, Fig. S4†). The lattice parameters of the parent, lithiated and fully delithiated phases are given in Table 3.

**Fig. 6:**
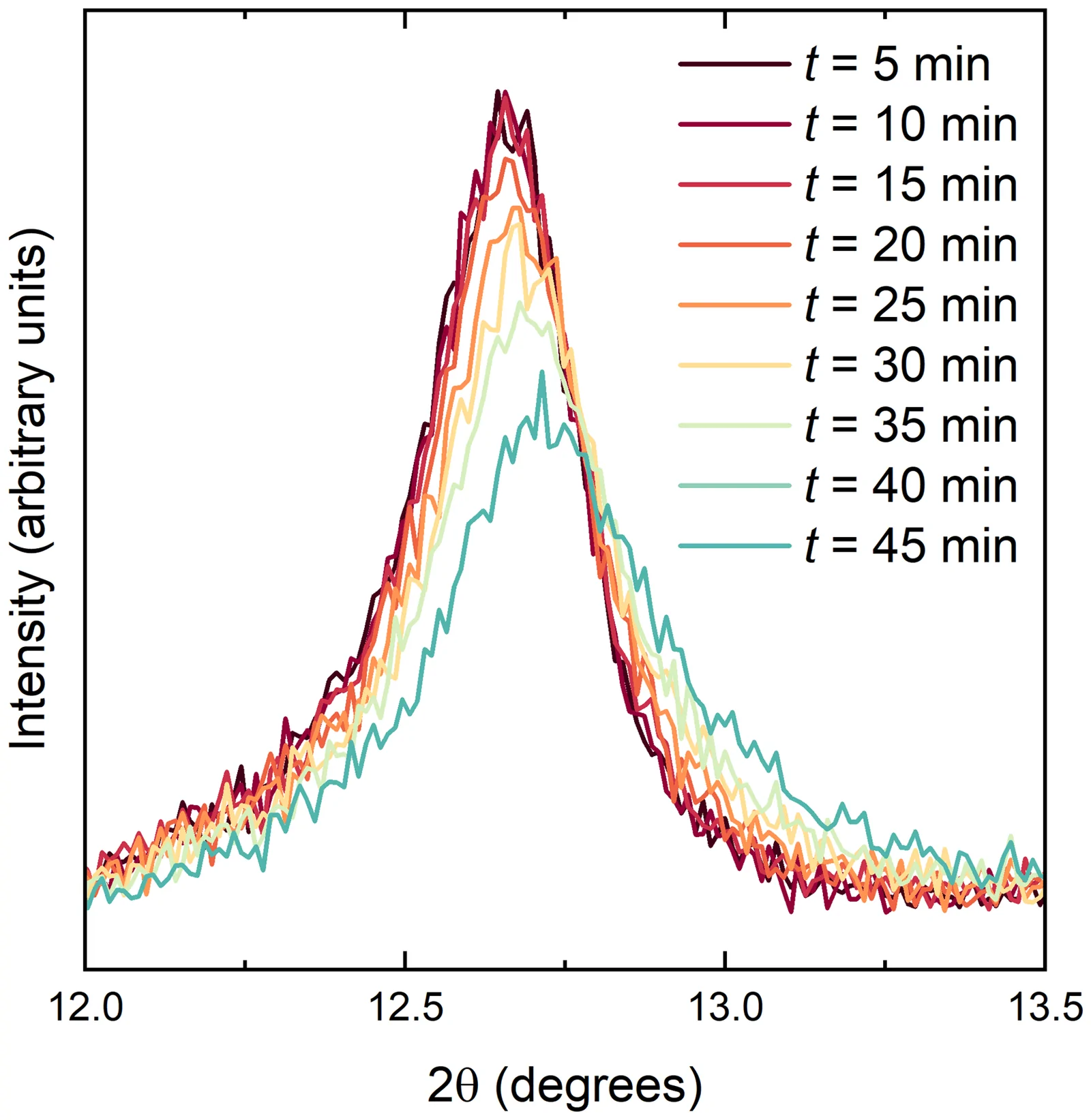
PXRD data (background subtracted) for Li_0.94_V_2_Te_2_O upon exposure to moist air.

**Table 3:** Lattice parameters for samples of V_2_Te_2_O before and after chemical lithiation and subsequent delithiation, obtained from Rietveld refinement of room-temperature laboratory PXRD data

Sample code	Description	*a* (Å)	*c* (Å)
NDK259	Original parent compound	3.9358(3)	13.247(2)
NDK260	Lithiated using BuLi → Li_0.94_V_2_Te_2_O^a^	4.0803(10)	13.987(4)
NDK260A	Delithiated in air	3.9264(3)	13.225(2)
NDK260B	Delithiated using H_2_O	3.9376(9)	13.248(4)

a Composition determined from powder neutron diffraction.

Lithium ions could also be extracted using water under anaerobic conditions. Small bubbles of gas were released during the reaction. Laboratory PXRD analysis on the product (ESI, Fig. S5†) indicated that V_2_Te_2_O had been re-formed, with the lattice parameters of the main *I*4/*mmm* phase decreasing again to values matching those of the initial parent compound (Table 3). Therefore, the proposed equation for Li deintercalation is:
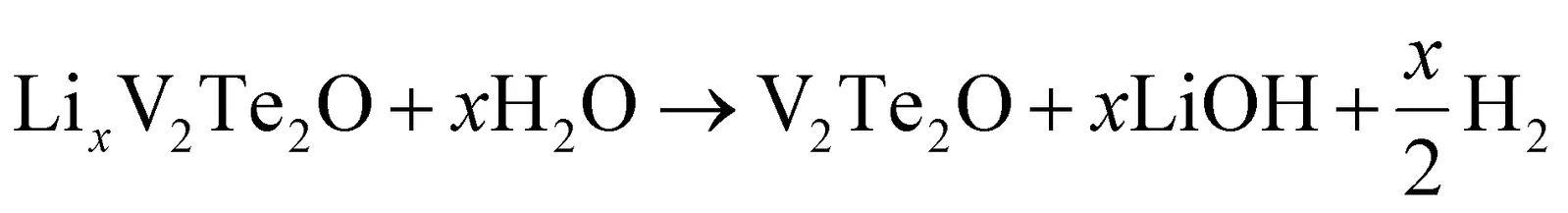
with LiOH being water-soluble and hence not present in the product PXRD pattern. Finally, reactions with a more oxidising solution of iodine in acetonitrile led to complete decomposition and oxidation of telluride ions to elemental tellurium.

### Electrochemical characterisation

Cyclic voltammetry data were collected from samples of V_2_Te_2_O in the range 1.0–3.7 V, as shown in Fig. 7(a). On sweeping the potential down in the first cycle, two reduction maxima were observed, the first between 1.7 and 1.4 V and the second between 1.4 and 1.0 V. Two distinct oxidation features were observed upon increasing the potential again at 1.2 to 1.8 V and 1.8 to 2.1 V. Upon cycling, the reduction features persisted (with gradually decreasing magnitude) but the oxidation features quickly became broader and by the fourth cycle had merged into a single wide feature situated between 1.2 and 2.5 V.

**Fig. 7:**
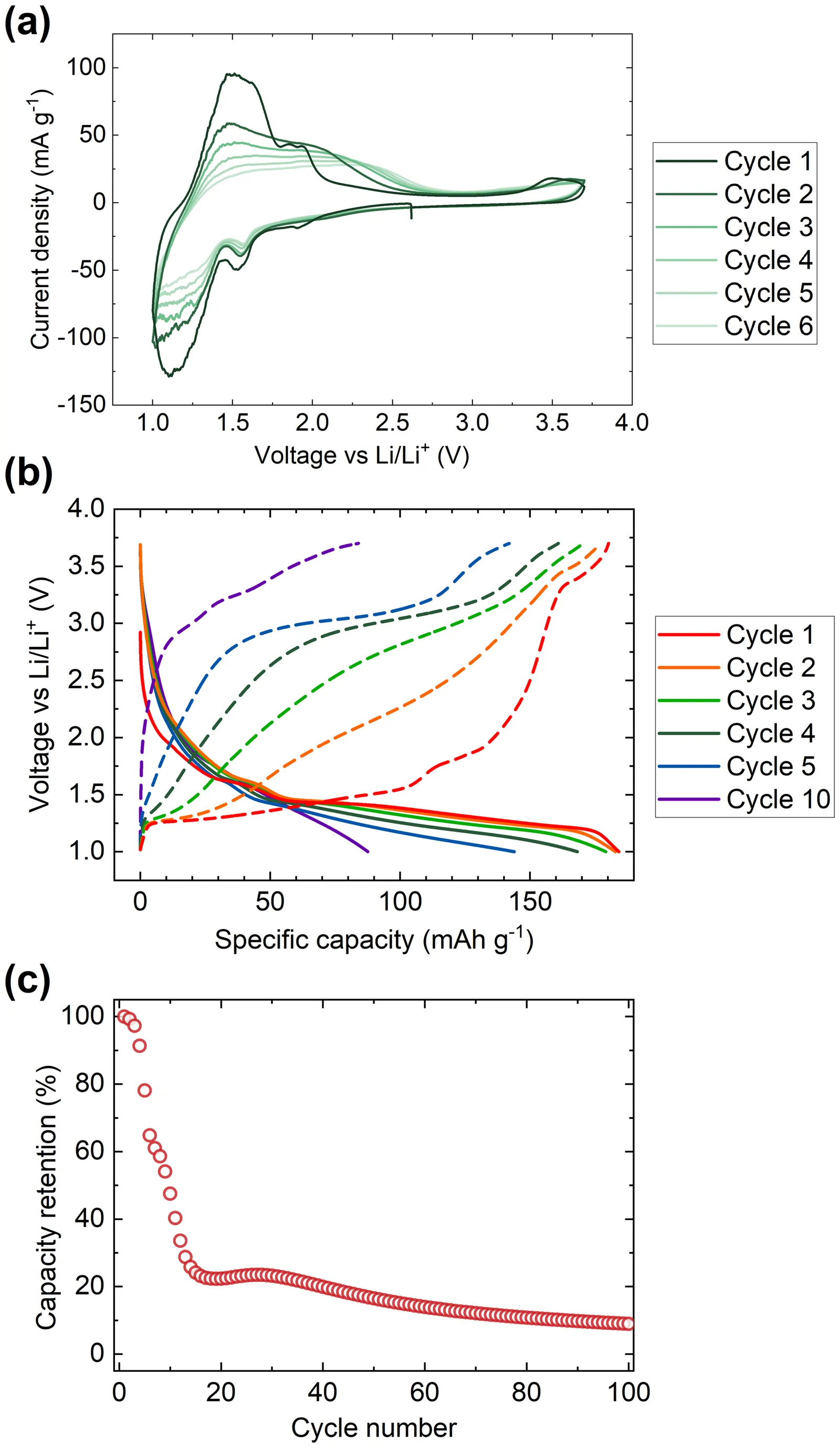
Electrochemical data for V_2_Te_2_O. (a) Cyclic voltammetry data, (b) discharge–charge cycling profiles, and (c) capacity data were collected in the range 1.0 to 3.7 V.

We assign the observed redox features to the reversible insertion and extraction of Li^+^ ions, as observed by chemical reduction and reoxidation. The broadening of the oxidation maxima and the overall decline in magnitude of both the reductive and oxidative processes are attributed to gradual decomposition of the cathode material. The loss of electrochemical activity upon cycling is also visible in the discharge–charge cycling data, Fig. 7(b and c). The initial discharge capacity of 180 mA h g^−1^ rapidly declines by more than 75% over the first 15 cycles then continues to tail off more slowly.

*Ex situ* PXRD was carried out on several cathode films after stopping the current and disassembling cells under argon at different points in the discharge–charge cycling profiles. The results are given in Table 4 and Fig. 8.

**Fig. 8:**
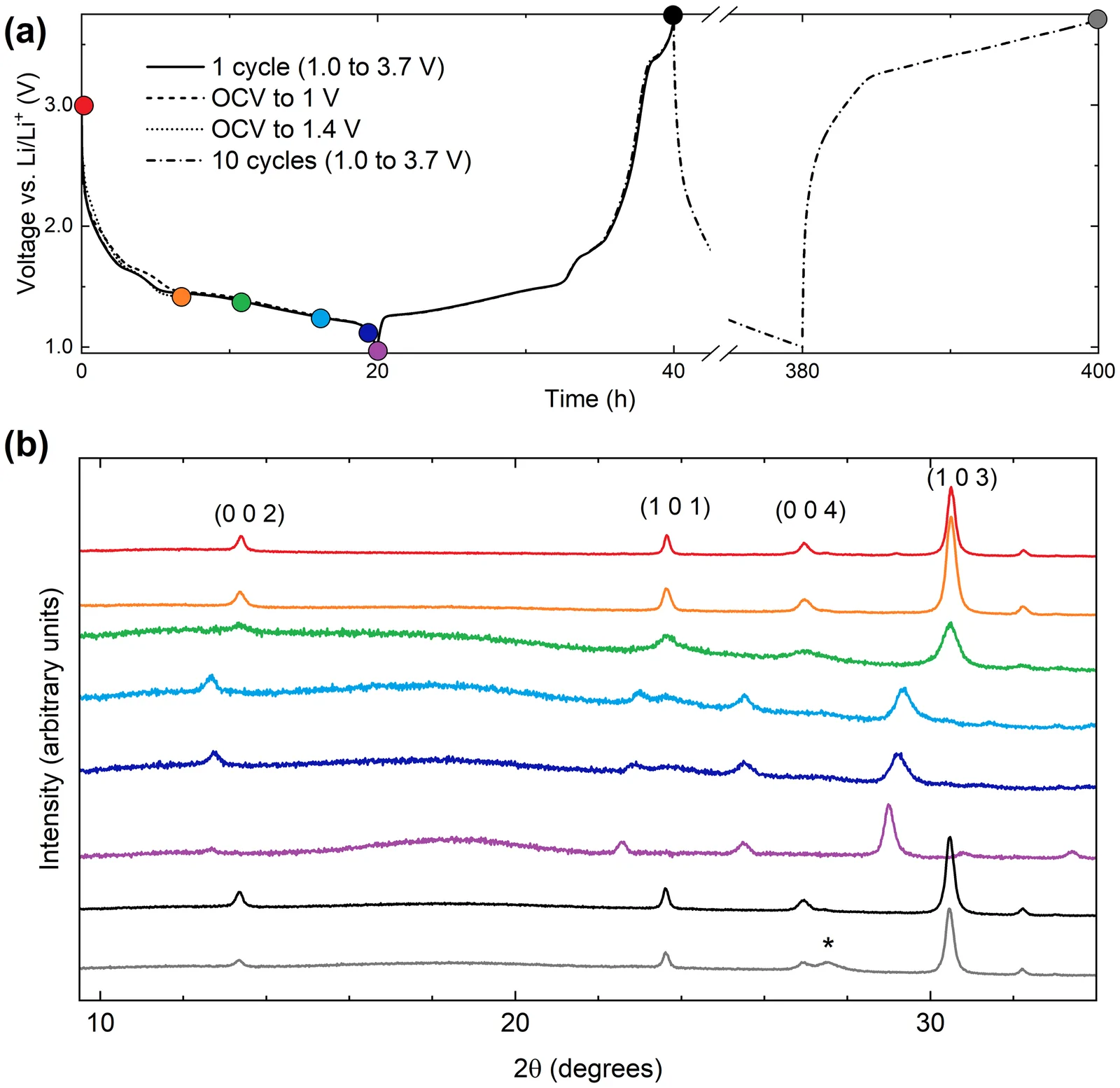
(a) Selected electrochemical charge–discharge cycling curves, (b) *ex situ* PXRD patterns with colours corresponding to the coloured circles in (a). The asterisk * indicates the main Bragg peak for elemental tellurium.

**Table 4:** Results of *ex situ* PXRD analysis on cycled cathode materials

Colour code in Fig. 8	Cycling state	*a* (Å)	*c* (Å)
Red	Open circuit voltage (OCV), rest for 2 h	3.9261(4)	13.227(2)
Orange	OCV, discharge to 1.4 V	3.9255(5)	13.223(2)
Green	OCV, discharge to 1.3 V	3.937(5)	13.27(2)
Light blue	OCV, discharge to 1.2 V	4.007(3)	13.93(2)
Dark blue	OCV, discharge to 1.1 V	4.057(6)	14.02(2)
Purple	OCV, discharge to 1.0 V	4.106(2)	13.959(9)
Black	1 discharge–charge cycle (1.0–3.7 V)	3.9256(4)	13.227(2)
Grey	10 discharge–charge cycles (1.0–3.7 V)	3.9158(6)	13.211(2)

The production of elemental Te (∼27 wt% after only 10 cycles, indicated by the * in Fig. 8(b)) indicates the oxidative decomposition of V_2_Te_2_O upon cycling *via* oxidation of telluride. The Te peak at 2*θ* = 27.5° is absent after the first discharge (purple dataset) and only appears after the first charge (black dataset), indicating that Te is produced in the oxidative part of the cycle. This is consistent with electronic structure calculations of the related oxytellurides Rb_0.8_V_2_Te_2_O and V_2_Te_2_O,^14,34^ which showed that the Te 5p bands are around 2 eV below the Fermi level and thus accessible at the voltages used here. The PXRD measurements also show that the reversible insertion and removal of lithium occurs mainly in the plateau between 1.4 and 1.0 V upon discharge and charge respectively.

Lattice parameters were obtained from Rietveld refinement of the *ex situ* cathode XRD data and have been plotted in Fig. 9. Visual comparison of these data with those in Fig. 4 show that, while the existing data does not allow the *x* axes to be calibrated quantitatively to each other, the electrochemical and chemical intercalation routes show similar behaviour in terms of both the relative rates of increase of *a* and *c* and their maximum values.

**Fig. 9:**
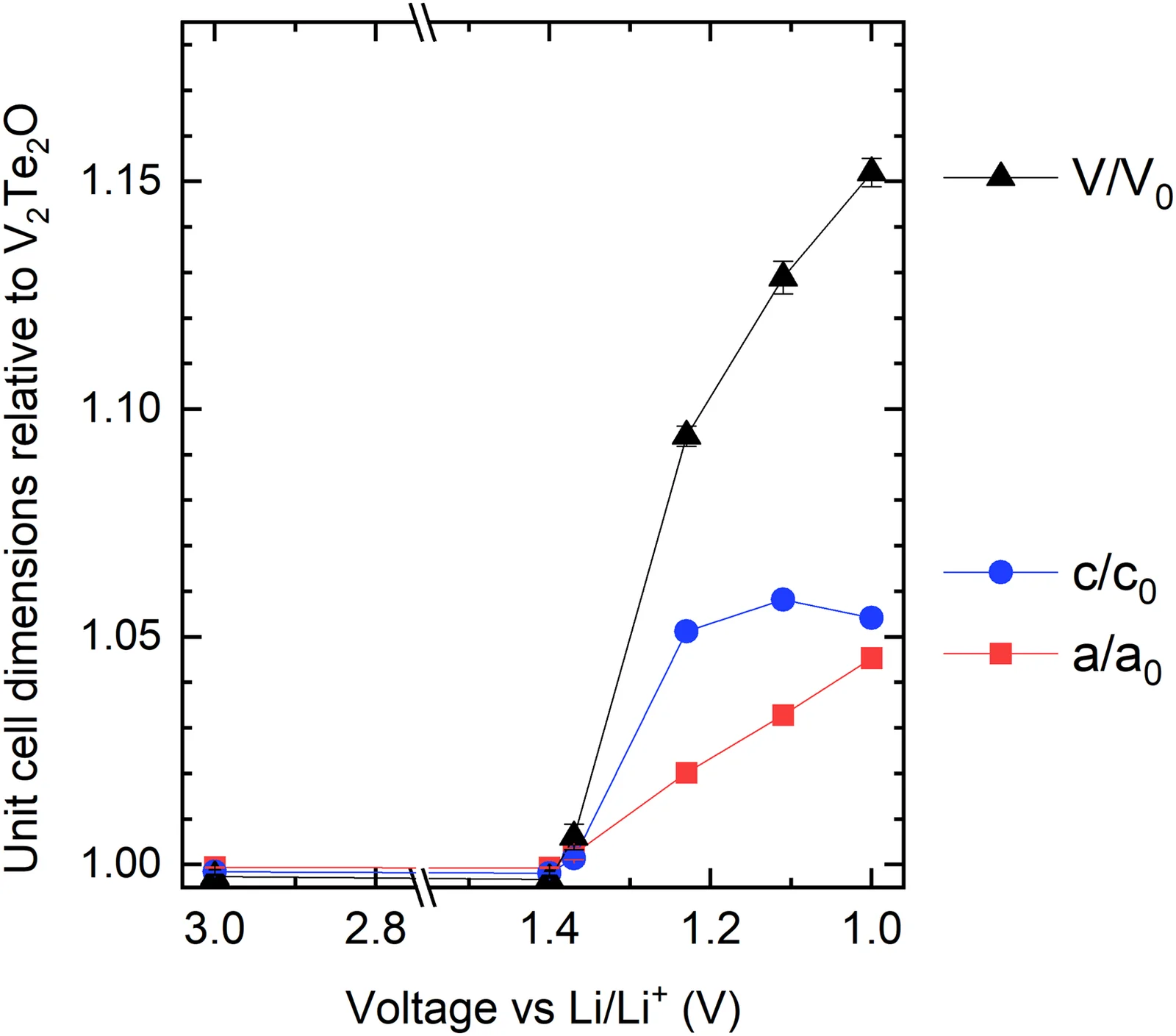
Refined lattice parameters for the electrochemically intercalated materials (cycled cathodes), relative to the published parameters for V_2_Te_2_O,^14^ as a function of the state of charge.

Insertion of 1 Li^+^ per V_2_Te_2_O has a theoretical specific capacity of 72 mA h g^−1^ so the initial capacity of 180 mA h g^−1^ would correspond to ∼2.5 Li. However, there are no changes to the V_2_Te_2_O lattice parameters between the open circuit voltage and 1.4 V (Table 4), which suggests that the first part of the cycle involves alternative reactions such as Li insertion into the conductive carbon (used to increase conductivity of the cathode) or irreversible conversion-type reactions. Fig. 7(b) shows that this would account for approximately the first 50 mA h g^−1^ of the capacity, leaving only around 1.8 Li to be inserted into the active material itself. This would tally with the slower rate of capacity fade after the capacity has dropped to ∼40 mA h g^−1^: the overall shape of the plot in Fig. 7(c) could represent V_2_Te_2_O/Li cycling with a rapid decline in capacity down to zero by 20 cycles, superimposed on a slower decline in the capacity from other parts of the cell.

## Discussion of the lithium composition: comparisons and limitations

In the chemical intercalation route, the refined Li occupancy for the sample analysed by PND was lower than expected for the amount of BuLi added, *e.g.* V_2_Te_2_O + 2*n*BuLi produced a refined composition Li_0.94_V_2_Te_2_O. We attribute this observation to side reactions which use up one or more molar equivalents of *n*BuLi. Full occupancy of the Li site would correspond to a composition of Li_2_V_2_Te_2_O and reduction to V^2+^. The fact that the maximum cell volume and *a* lattice parameter were observed when 3 or 4 molar equivalents of BuLi were used in the chemical synthesis (*i.e.* excesses of Li compared with the availability of intercalation sites and reasonable vanadium redox chemistry (Fig. 4)), confirms this. In the electrochemical intercalation route, the initial invariance of the lattice parameters of the oxytelluride phase at early stages of discharge suggests side reactions, perhaps related to the conductive carbon in the cathode mixture. The current passed in the later portion of the discharge, during which there is a change in the lattice parameters, corresponds to the insertion of 1.8 moles of Li per mole of V_2_Te_2_O on discharge to 1.0 V, and accordingly the increase in cell volume of the most lithiated phase, formed by discharging to 1.0 V (purple dataset in Fig. 8) is about 10% less than the maximum found using excess *n*BuLi in the chemical route, consistent with the chemical route giving a limiting composition of Li_2_V_2_Te_2_O. The lattice parameters derived from cathodes at intermediate states of charge map well onto those of the chemically intercalated samples made with 0.5–2 equiv. *n*BuLi.

The low weight of Li and the fact that the variation in cell volume may not be linear with composition over the full compositional range means that it is not feasible to calibrate the Li contents directly between the chemically and electrochemically intercalated routes without an extensive neutron diffraction study on several different samples, including large samples of electrochemically cycled materials, which is beyond the scope of the present work. However, our assumption of ∼50 mA h g^−1^ of initial capacity from side processes would suggest ∼1.8 Li as the limiting composition upon electrochemical discharge *vs*. Li/Li^+^ down to 1.0 V, and the chemical route is able to access slightly higher Li contents based on the slightly larger maximum unit cell volume.

A composition of Li_2_V_2_Te_2_O would correspond to an average vanadium oxidation state of +2 which is the lowest stable non-zero oxidation state. However, in other compounds containing the same [M_2_Q_2_O] layer, where M is a 3d transition metal and Q is a chalcogenide (S, Se, Te) or pnictide (As, Sb, Bi) ion, we note that there is a trend for early transition metals to favour higher oxidation states than later transition metals, in line with the trend in ionisation energies across the series. For example, the Ti oxypnictides (such as BaTi_2_Sb_2_O) all contain Ti^3+^ (ref. 37) and the Ti and V oxychalcogenides exhibit metal oxidation states between +2.5 and +3;^15^ the chromium oxyarsenide Sr_2_CrO_2_Cr_2_OAs_2_ contains Cr^3+^ in the [M_2_Q_2_O] layer.^38^ However, for the later transition metals Mn, Fe and Co there are a number of known oxychalcogenides which all contain M^2+^ in the [M_2_Q_2_O] layer.^39–42^ In our sample probed by neutron diffraction, with a relatively low level of impurities, the vanadium ions reached a minimum oxidation state of about +2.5. Further reduction is possible chemically or electrochemically based on the lattice parameters in Fig. 4 and 9, however the increasing prevalence of side products on further reduction may be a consequence of the difficulty in attaining the V^2+^ oxidation state. Given the uncertainties in the refined parameters and the evident presence of side reactions, additional neutron diffraction experiments on samples made with a larger excess of *n*BuLi would be required to confirm whether the proposed limiting composition of Li_2_V_2_Te_2_O can be attained. Furthermore, neutron diffraction on an *in situ* electrochemical cell would be invaluable because it would provide an accurate limiting composition for the electrochemical intercalation route, but could also act as a calibration curve between Li content and lattice parameters.

## Conclusions

The van der Waals layered oxytelluride V_2_Te_2_O was investigated as a host for lithium intercalation reactions. Chemical intercalation using *n*-BuLi resulted in the formation of Li_*x*_V_2_Te_2_O with an expanded unit cell but no significant change to the host crystal structure. Powder neutron diffraction was used to determine the composition and crystal structure of a representative sample, in which Li occupies a six-coordinate site bonded to one oxide and five telluride anions. This sample had *x* = 0.94(2), and examination of powder X-ray diffraction data for various samples suggests that the maximum extent of chemical lithiation *via* this route approaches *x* = 2. The intercalated Li^+^ ions could subsequently be fully deintercalated using air or water. Electrochemical investigations, including *ex situ* structural analysis at different points during the cycling process, reinforce the conclusion of reversible lithiation of V_2_Te_2_O (again up to a limiting composition, here *x* ∼1.8 when the cell is discharged to 1.0 V) albeit with significant capacity fade on cycling, owing to gradual decomposition which includes oxidation of telluride ions to elemental Te during the charging process. Future work will focus on *in situ* and *ex situ* X-ray and neutron diffraction experiments in order to calibrate the Li content and lattice parameters across both the chemical and electrochemical intercalation routes with changes in physical properties as a function of vanadium oxidation state.

## Author contributions

N. D. K. devised the project, carried out the synthesis, the crystallographic characterisation and the physical property measurements. H. G. performed the electrochemical cycling experiments and analysis and K. M. S. performed powder diffraction experiments on the electrochemically synthesised samples. I. da S. collected and processed the powder neutron diffraction data. S. J. C. directed the research and provided funding and resources. N. D. K. wrote the paper with input from S. J. C. and the other authors.

## Conflicts of interest

There are no conflicts of interest to declare.

## Supplementary Material

DT-054-D5DT00159E-s001

## Data Availability

The data used in this publication are available from the author on reasonable request. The neutron powder diffraction data are available through the following link. https://doi.org/10.5286/ISIS.E.RB2390120-1.
