# Supplementary material for: Chemical and electrochemical lithiation of van der Waals oxytelluride V2Te2O
Source: Dalton Trans. 2025 Feb 21;54(20):8123–32. doi: 10.1039/d5dt00159e (PMC13725693; doi:10.1039/d5dt00159e)
Supplement: Supplementary file 1 [file DT-054-D5DT00159E-s001.pdf]

Supplementary Information (SI) for Dalton Transactions.
This journal is © The Royal Society of Chemistry 2025

ELECTRONIC SUPPLEMENTAL INFORMATION

Chemical and Electrochemical Lithiation of Van Der Waals Oxytelluride V_2Te_2O

Nicola D. Kelly[1,2,#], Heather Grievson[2,3], Katherine M. Steele[1], Ivan da Silva[4] and Simon J. Clarke[1,2]*

[1] Department of Chemistry, University of Oxford, Inorganic Chemistry Laboratory, South Parks Road, Oxford, OX1 3QR, UK.

[2] The Faraday Institution, Quad One, Harwell Campus, Didcot, OX11 0RA, UK.

[3] Department of Materials Science and Engineering, University of Sheffield, Sir Robert Hadfield Building, Sheffield, S1 3JD, UK.

[4] ISIS Facility, STFC Rutherford Appleton Laboratory, Harwell Campus, Didcot, OX11 0QX, UK.

# Present address: Jesus College, University of Cambridge, CB5 8BL, UK.

* simon.clarke@chem.ox.ac.uk

Neutron refinements

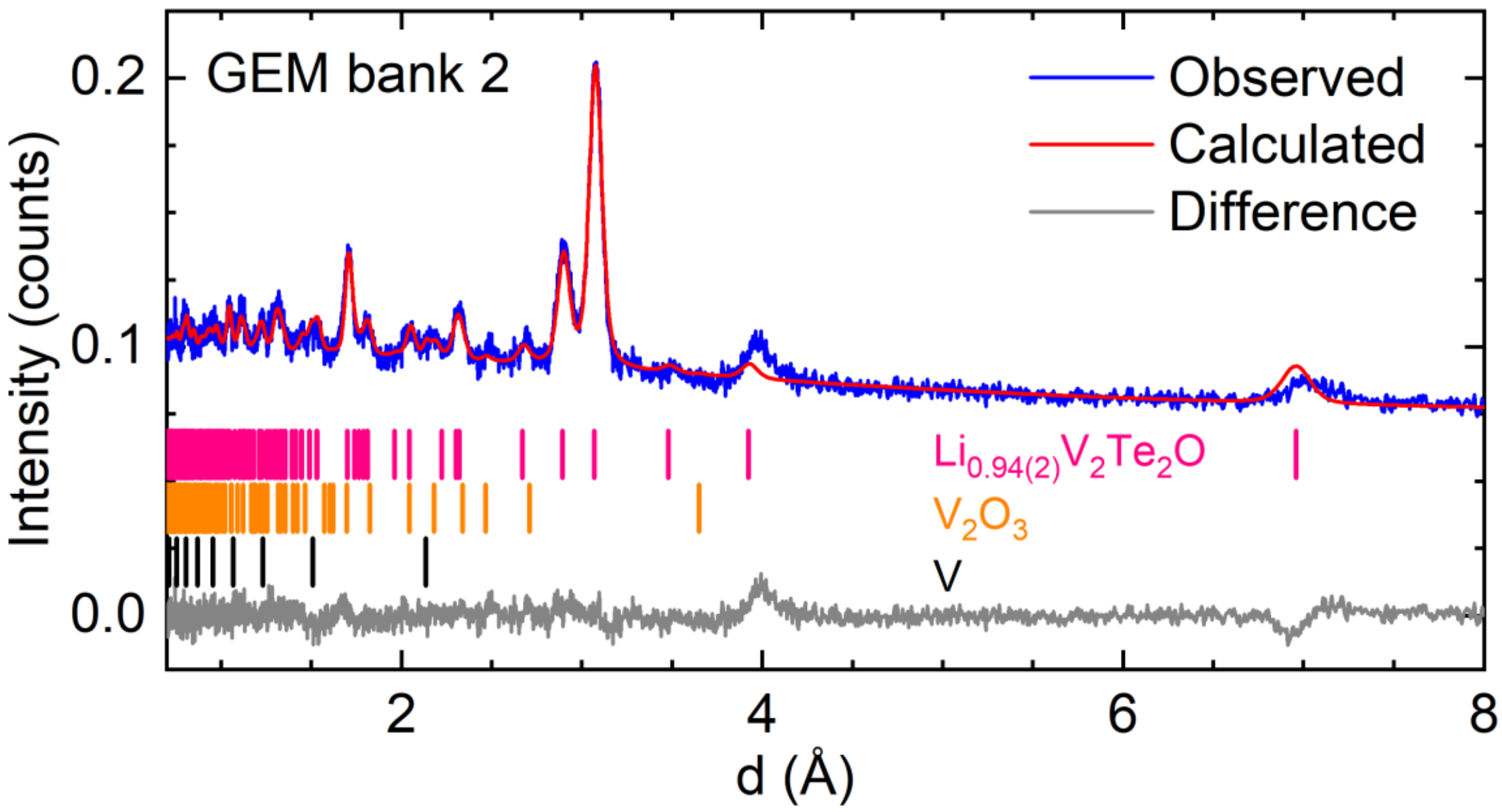

Figure S1: Rietveld refinement of $Li_{0.94}V_2Te_2O$ against GEM bank 2 data. Blue – experimental data; red – calculated intensities; grey – difference pattern; pink tick marks – Bragg reflection positions for $Li_{0.94}V_2Te_2O$ (pink), V_2O_3 (orange) and V (black).

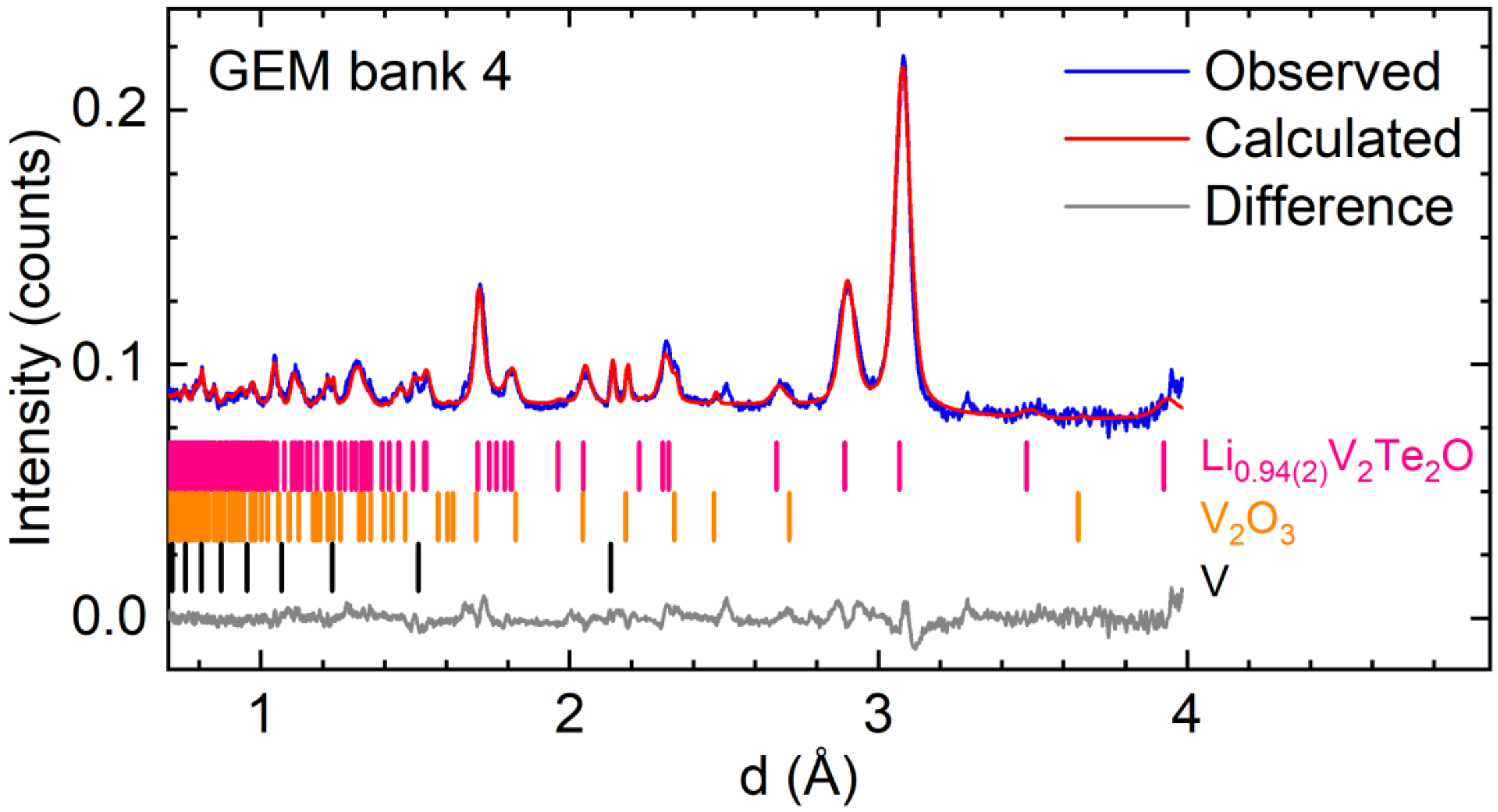

Figure S2: Rietveld refinement of $Li_{0.94}V_2Te_2O$ against GEM bank 4 data. Blue – experimental data; red – calculated intensities; grey – difference pattern; pink tick marks – Bragg reflection positions for $Li_{0.94}V_2Te_2O$ (pink), V_2O_3 (orange) and V (black).

Impurity phase observed by PXRD

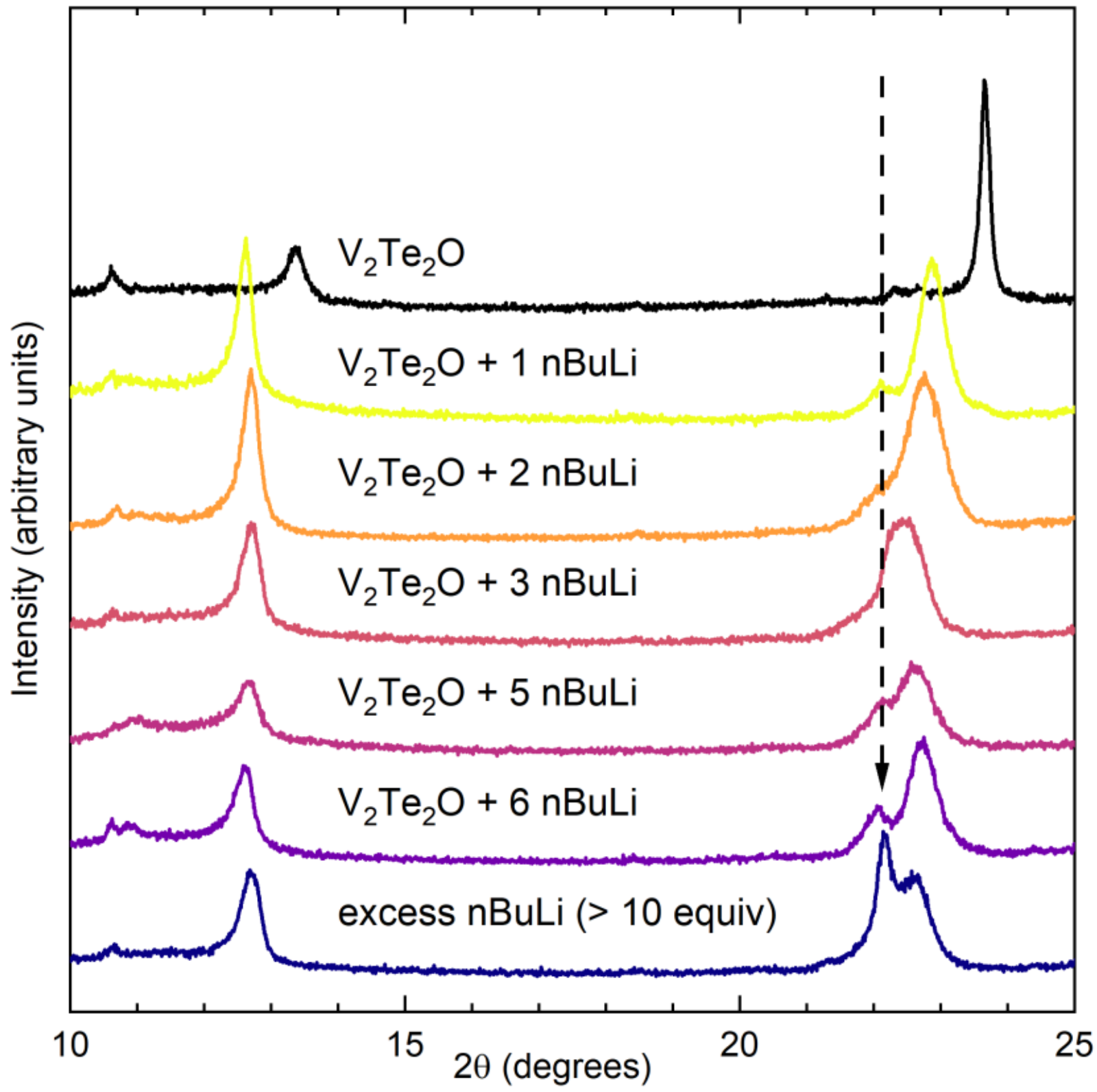

Figure S3: Laboratory PXRD patterns for intercalated samples, showing the increasing amounts of an unindexed secondary phase (peak at d ~ 4 Å) with increasing amounts of nBuLi.

Air exposure PXRD

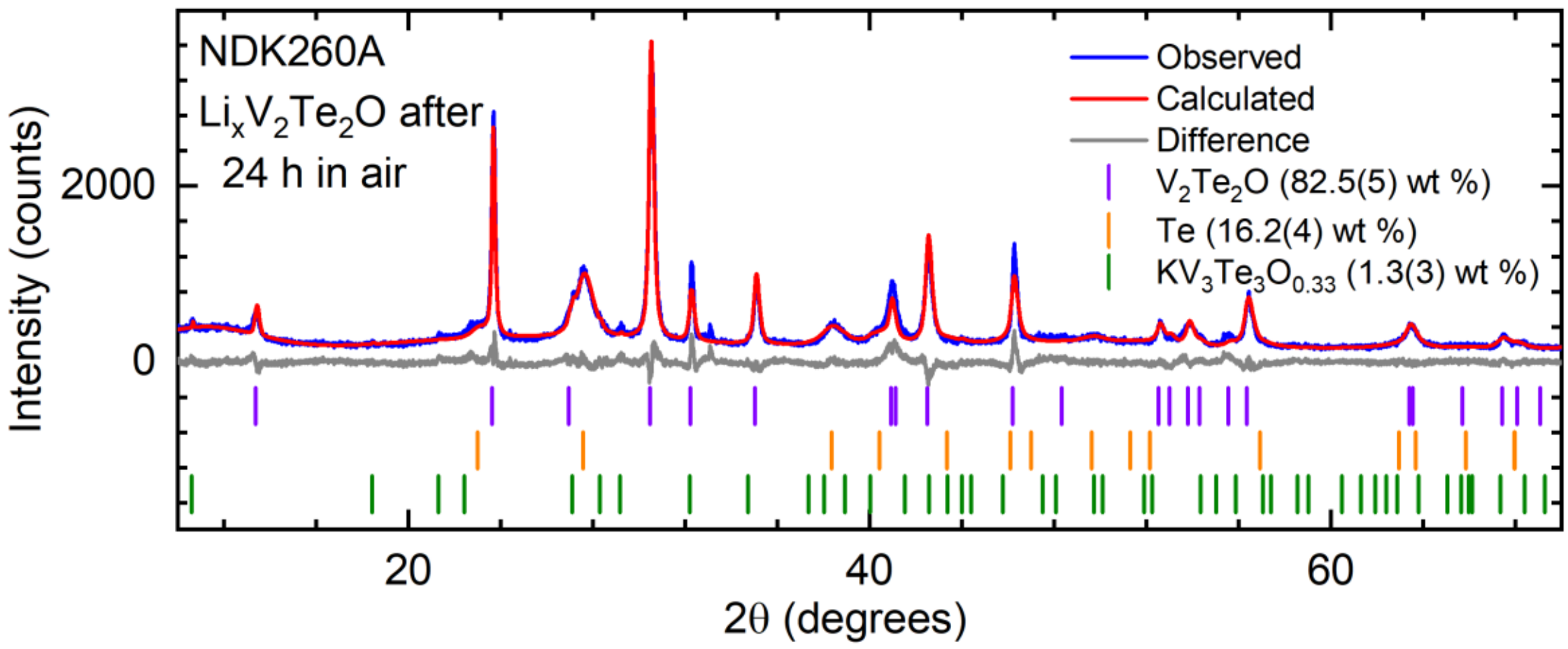

Figure S4: Rietveld refinement of laboratory PXRD data for $Li_{0.94}V_2Te_2O$, 24 h after exposure to moist air. The sample had fully decomposed into V_2Te_2O + Te.

Water reaction PXRD

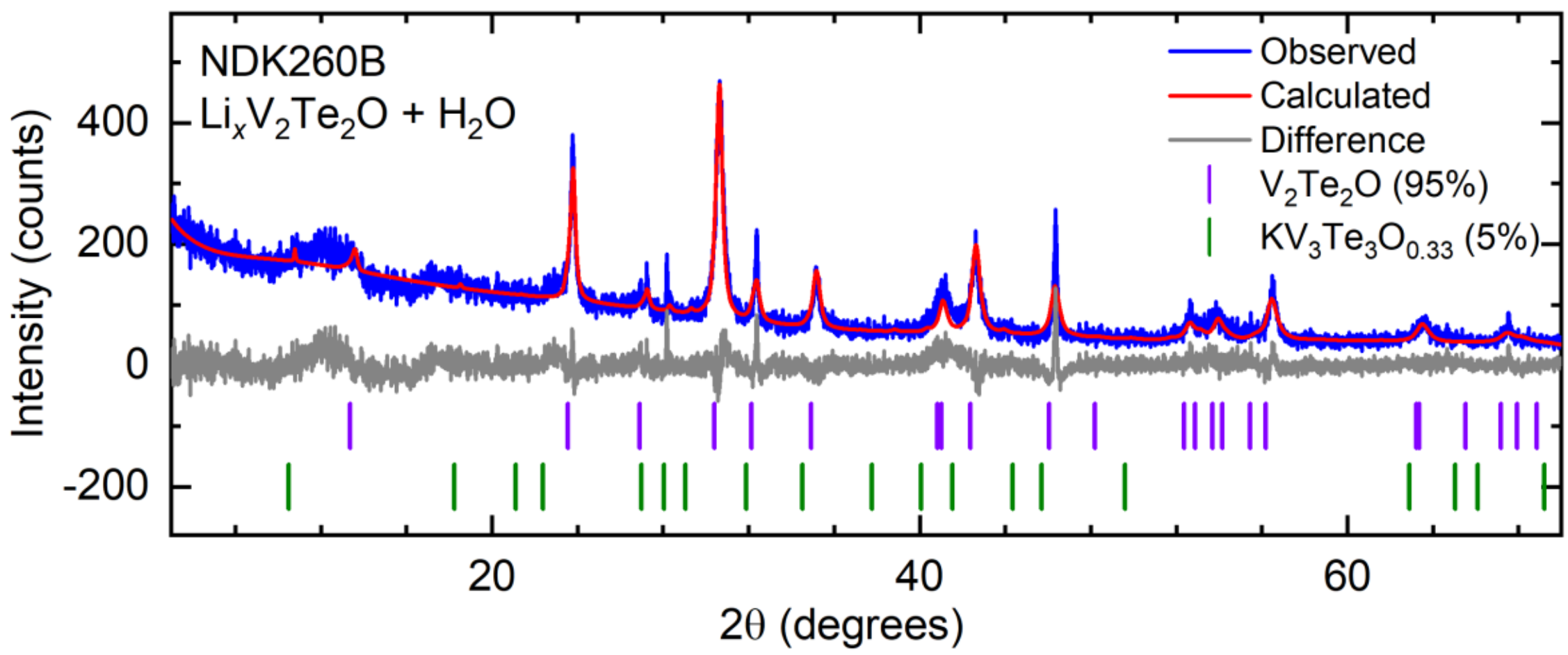

Figure S5: Rietveld refinement of laboratory PXRD data for the product of the reaction $Li_{0.94}V_2Te_2O$ + H_2O (under inert atmosphere).

Magnetometry

Magnetic measurements were made using a Quantum Design MPMS-XL or MPMS-3 SQUID magnetometer. A powder sample of mass 20–30 mg (accurately measured in each case) was placed in a gelatine capsule inside a plastic straw as the sample holder. The susceptibility $\chi \approx M/H$ was determined by measuring the magnetic moment (M) on warming from 5–350 K in an applied field (H) of 100 Oe after cooling in the applied field (FC) or in zero field (ZFC). A magnetisation hysteresis loop was measured as a function of applied field in the range ± 5 T at temperatures of 5 and 300 K.

The susceptibility curves for the lithiated samples (Figure S6) increase sharply below T_C ~ 40 K, indicating ferromagnetic (FM) interactions. The $M(H)$ data at low temperature show soft ferromagnetism with a very small hysteresis. However, for several reasons we believe that this may originate from the unidentified impurity phase with its main diffraction peak at d ~ 4 Å. The maximum magnetisation (at T ~ 30 K) is only 5 x 10^{-4} μ_B per formula unit of $Li_xV_2Te_2O$ and the susceptibility drops again below 30 K rather than continuing to increase upon cooling, which suggests that the FM fraction of the total sample is small. Furthermore, the onset temperature of the transition is identical across samples made using different amounts of nBuLi, whereas different values of x in $Li_xV_2Te_2O$ would be expected to produce different electron counts and thus different T_C's. At 100–350 K the susceptibility slowly decreases in a non-Curie-Weiss fashion and the absolute values of the magnetisation are of the same order as powder samples of the parent material V_2Te_2O. The behaviour is consistent with Pauli paramagnetic behaviour across the compositional range, as per the parent V_2Te_2O [1].

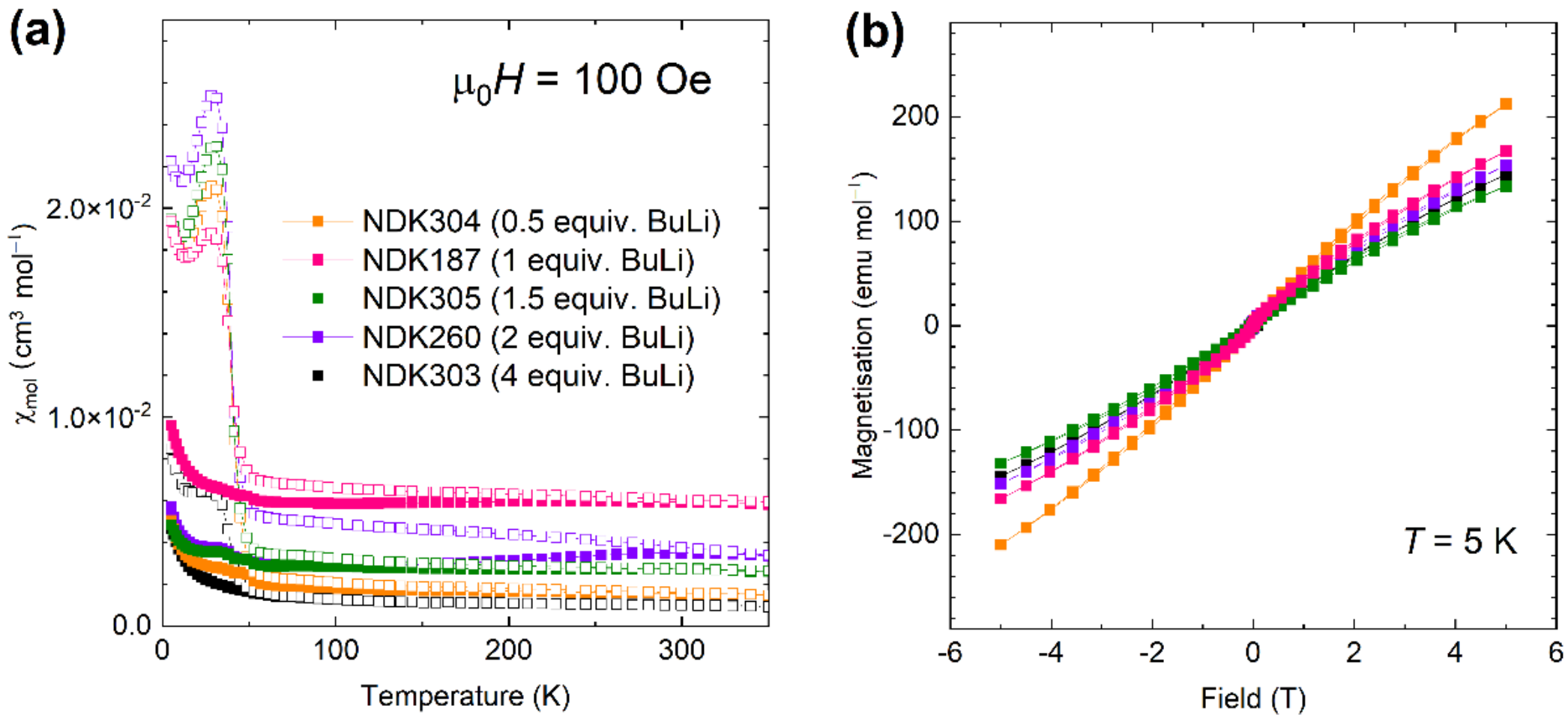

Figure S6: Magnetic data for $Li_xV_2Te_2O$ samples. (a) Magnetic susceptibility at 100 Oe measured in the ZFC (filled symbols) and FC (open symbols) regimes. (b) Hysteresis loop at 5 K.

---

[1] A. Ablimit *et al.*, "V_2Te_2O: A Two-Dimensional van der Waals Correlated Metal," *Inorg. Chem.*, vol. 57, no. 23, pp. 14617–14623, 2018.
